# Melatonin supplementation alleviates cellular damage and physical performance decline induced by an intensive training period in professional soccer players

**DOI:** 10.1371/journal.pone.0273719

**Published:** 2022-09-02

**Authors:** Mohamed Amine Farjallah, Kais Ghattassi, Anis Kamoun, Ahmed Graja, Lobna Ben Mahmoud, Tarak Driss, Kamel Jamoussi, Zouheir Sahnoun, Nizar Souissi, Piotr Zmijewski, Omar Hammouda

**Affiliations:** 1 Faculty of Medicine, Research Laboratory, Molecular Bases of Human Pathology, LR19ES13, University of Sfax, Sfax, Tunisia; 2 “Physical Activity, Sport and Health” Research Unit, UR18JS01, National Sport Observatory, Tunis, Tunisia; 3 Research Unit, Education, Motricity, Sport and Health, UR15JS01, High Institute of Sport and Physical Education, University of Sfax, Tunisia; 4 Research Laboratory of Evaluation and Management of Musculoskeletal System Pathologies, LR20ES09 University of Sfax, Sfax Tunisia; 5 Faculty of Medicine, Department of Pharmacology, University of Sfax, Sfax, Tunisia; 6 Interdisciplinary Laboratory in Neurosciences, Physiology and Psychology: Physical Activity, Health and Learning (LINP2-APSA), UPL, Paris Nanterre University, UFR STAPS, Nanterre, France; 7 Biochemistry Laboratory, CHU Hedi Chaker, University of Sfax, Sfax, Tunisia; 8 Jozef Pilsudski University of Physical Education in Warsaw, Warsaw, Poland; Poznan University of Physical Education, POLAND

## Abstract

Melatonin has been proved to have positive effects on cellular damage and metabolic regulation. The aim of the study was to determine the effect of melatonin supplementation during an intensive training period on physical performance decline, oxidative stress and cellular damage state. The investigation was conducted on 20 soccer players who participated in an exhaustive six-day training schedule associated with daily 5 mg oral melatonin or placebo ingestion. Resting blood samples and physical performance were measured before and after the training period. The mixed 2-way ANOVA (group x training camp) showed that compared to placebo, melatonin intake prevented an increase in advanced oxidation protein products (p>0.05) and increased the antioxidant enzyme activity (i.e., superoxide dismutase; p<0.001). In addition, melatonin prevented an increase of biomarkers of renal function (e.g., creatinine; p>0.05) and biomarkers of muscle (e.g., creatine kinase; p>0.05) and liver (e.g., gamma-glutamyltransferase; p>0.05) damage. Furthermore, melatonin alleviated the deterioration in physical performance (countermovement jump, five-jump test and 20-m sprint; p>0.05). In conclusion, the obtained data showed increased oxidative stress and renal, muscle and liver damage in professional soccer players during an exhaustive training schedule. Melatonin intake during the training period exerts beneficial effects on physical performance and protects tissues against the deleterious effects of reactive oxygen species and cellular damage.

## Introduction

Skeletal muscle may undergo cellular damage especially in response to eccentric and exhaustive exercise [1]. It often persists for many days after finishing exercise and symptoms mostly include increased reactive oxygen species (ROS), disruption of muscle fiber structures and integrity, release of intra-myocellular proteins into the circulation and elevations in markers of inflammation and various interleukins [2]. In addition, cellular damage is associated with muscle pain, localized swelling and altered joint kinematics, and may seriously limit physical performance during training period and/or competition [2].

Therefore, various therapeutic and recovery strategies have been investigated and widely employed to alleviate the impact of exercise-induced cellular damage on physical performance [3,4]. Among them, supplementation has been purported as effective to help athletes tolerate increased training loads and demanding competitive schedules [3,5,6]. It may help to treat or prevent nutritional deficiencies, and have an important ergogenic effect [6].

An optimal supplement would be an agent that simultaneously exhibits antioxidant and anti-inflammatory properties, as well as having potential favorable effects on physical fitness [6]. A molecule which meets these conditions is melatonin (MEL; N-acetyl-5-methoxytryptamine). This indoleamine is mainly produced by the pineal gland at night and is responsible for the synchronization of various physiological processes [7]. It directly scavenges ROS and protects against lipid peroxidation, protein oxidation and DNA damage [8]. It manifests indirect antioxidant properties by stimulating the enzymatic antioxidant system and producing molecules protecting against the deleterious effects of oxidative stress [8,9]. Furthermore, it reveals promising results in the prevention and reduction of inflammation [8,10]. Likewise, it reduces muscle damage [8,10] and has a beneficial impact on skeletal muscle healing and tissue repair [11]. Moreover, it facilitates muscle adaptation to physical exercise [12] and shows favorable effects on aerobic tolerance and physical performance [8,13].

Antioxidant status and serum levels of muscle and liver enzymes are biomarkers of the functional status of the body, which may be stressed following intensive training as a consequence of both metabolic and mechanical factors [14]. Knowing that MEL could act in many ways, it is very important to analyze several biochemical and metabolic parameters to identify the effect of this indole on athletes’ health during an intensive training period. Therefore, the aim of the present study was to investigate the effect of MEL ingestion on physical performance decline and biochemical and metabolic responses during an intensive training period for soccer players. We hypothesized that MEL could exert a protective effect by alleviating the exercise-induced oxidative stress and muscle damage. In addition, MEL could have a beneficial effect on physical performance and therefore could be used as a therapeutic strategy capable of helping athletes in congested schedules.

## Materials and methods

### Participants

The sample size was calculated and established using the G*Power 3 software [15] and α and power were fixed at 0.05 and 0.80, respectively. Based on a similar previous study [13] and discussions between the authors, effect size was estimated to be 0.6 (medium effect). The appropriate sample size was 17 participants. However, to anticipate possible drop-out of some participants, 24 professional soccer players from a Tunisian first league team were recruited. Four participants dropped out of the study: one participant was absent during the second testing session, one participant was injured and two participants withdrew because they refused blood collection. Thus, a total of 20 soccer players [age: 18.8 ± 1.3 years, body mass: 70.0 ± 10.6 kg, body height: 181 ± 8 cm, BMI: 21.27 ± 1.87 kg/m^2^; mean ± standard deviation (SD)] from a first league team participated in the present study. All participants were evaluated by routine clinical laboratory tests and standard physical examination. They were all males, healthy, non-smokers and without significant medical contraindications. They were not taking any supplementation or medication, and they did not do any transmeridian travel in the last month.

All subjects were made aware of the course of the study and provided their informed written consent before participation. The experimental design of the study conformed to the bioethical principles of the Declaration of Helsinki and was approved by the club and by the local Institutional Review Board (i.e., the Personal Protection Committee). The reference is: CPP SUD N° 0185/2019.

### Experimental design

All the included athletes were participating in a 6-day training camp (TC) in the preparatory phase of the sport season. During this training period, players lived in the sports complex of the club in order to control their food intake and sleep schedules (i.e., bedtime: 10:30 ± 00:30 p.m., wake-up time: 06:30 ± 00:30 a.m.). Moreover, they were training twice a day (i.e., morning sessions started at 10:30 a.m. and the afternoon sessions at 5:30 p.m.). On the first day, participants came to the laboratory at 7:00 a.m. and resting blood samples (10 ml) were taken from an antecubital vein in a fasted state. At 10:00 a.m., participants started their usual team warm-up, then they performed a battery of physical tests before doing their first training session. The testing battery was performed in the following order: squat jump (SJ), countermovement jump (CMJ), five-jump test (5-JT), modified agility T-test (MAT) and 20-m sprint (20m-Sp). Physical tests were performed as described by Farjallah et al. [16] and Cheikh et al. [13].

A simple randomization procedure was used to classify the participants into two distinct homogenized groups: the MEL group (n = 10) and the placebo group (n = 10). The anthropometric and physical characteristic of each group are summarized in Table 1. Maximum aerobic speed had been measured 1 week before the TC, with the Yo-Yo intermittent recovery test level-1 [17].

**Table 1:** Anthropometric and physical characteristics of melatonin (MEL) and placebo (PLA) groups.

	PLA	MEL	t	p
**Age (years)**	18.9 ± 0.4	18.8 ± 0.4	0.38	0.71
**Body mass (kg)**	70.9 ± 10.6	69.3 ± 6.8	0.35	0.73
**Body height (cm)**	181 ± 11	182 ± 6	0.13	0.89
**BMI (kg/m** ^**2**^ **)**	21.58 ± 2.05	21.03 ± 1.87	0.56	0.59
**Maximum aerobic speed (km/h)**	16.43 ± 0.67	16.50 ± 0.61	0.22	0.83

During the six days of the TC, participants of the two groups took one capsule of 5 mg of MEL (Jamieson Laboratories Toronto, Montreal, Canada) or placebo (PLA) composed of lactose, starch, and cellulose. Supplements were taken daily at 7:00 p.m. This investigation was a randomized double blind parallel group study. Neither staff nor participants were informed about the composition of the two capsules, and blinding was rigorously maintained by emphasizing to staff and participants that both capsules adhered to healthy principles and that each capsule was endorsed by many sports medicine experts. After the training period, the testing battery and the resting blood samples were repeated in the same order, at the same time of day and in the same conditions.

### Blood sampling and variables

Fasting blood samples were collected from a forearm vein after 10 min of seated rest. An EDTA tube was used to determine oxidative stress parameters such as advanced oxidation protein products (AOPP) and superoxide dismutase (SOD). In addition, a heparinized tube was used to measure creatine kinase (CK), gamma-glutamyltransferase (GGT), alkaline phosphatase (AP), creatinine (Cre), urea (Ur), total protein (TP), total cholesterol (TCh), high-density lipoprotein (HDL), low-density lipoprotein (LDL), triglyceride (Tg) and glucose (Gl). Tubes were placed in an ice bath. Then they were centrifuged immediately at 2500 × g and 4°C for 10 min.

Aliquots of the resulting plasma were stored at -80°C until analyzed. To eliminate inter-assay variance, all samples were analyzed in the same assay run. All assays were performed in duplicate in the same laboratory with simultaneous use of a control serum from Randox.

### Analysis of blood variables

AOPP levels were determined by the method of Kayali et al. [18]. Plasma was treated with phosphate buffer (0.1 M; pH 7.4). After 2 min incubation, 1.16 M potassium iodide and 10% TCA were added to the mixture. The concentration of AOPP for each sample was calculated based on an extinction coefficient of 261 M^-1^.cm^-1^ at 340 nm.

SOD activity was assayed in terms of its ability to inhibit the photochemical reduction of nitro blue tetrazolium (NBT) according to Beauchamp and Fridovich [19]. The reaction mixture contained 0.1 M potassium phosphate buffer, 0.26 mM riboflavin, 2.69 mM methionine and 2.64 mM NBT, with suitably diluted plasma in a total volume of 1.5 ml. The assay mixture was illuminated for 20 min by a 20 W fluorescent lamp, in an aluminium foil-lined container. Illumination of riboflavin in the presence of O_2_ and an electron donor such as methionine generates superoxide anions. The reduction of NBT by superoxide radicals to blue colored formazan was followed at 580 nm. A control without the enzyme source was always included. One unit of SOD activity is defined as the amount of enzyme that inhibits the rate of NBT reduction by 50% under the specified conditions.

CK, GGT, AP, Cre, Ur, TP, TCh, HDL, LDL, Tg and Gl measurements were done as adapted for the autoanalyzer by the Siemens ADVIA 1800 chemistry system (Erlangen, Germany).

CK activity was determined with the N-acetyl-L-cysteine method by measuring nicotinamide adenine dinucleotide phosphate formed by hexokinase and the D-glucose-6-phosphate dehydrogenase coupled enzymatic system. N-acetyl-L-cysteine is used as the activator of CK. The intra-assay coefficient of variation for this parameter kit was 1.85%. GGT activity was determined spectrophotometrically by the enzymatic method using L-gammaglutamyl-3-carboxy-4 nitroanilide as a donor substrate and glycylglycine as an acceptor substrate. The intra-assay CV for the gamma-glutamyl kit was 2.3. AP activity was determined spectrophotometrically by the hydrolysis of phosphate paranitrophenyl. The intra-assay CV for the AP kit was 4.6%. Cre concentrations were determined by the Jaffé method [20]. The assay is based on the reaction of Cre with sodium picrate. Ur was determined using an enzymatic method. The intra-assay CV was 0.33%. TP, TCh, Tg and Gl were determined using an enzymatic colorimetric method. The intra-assay CV values for these parameters were <7%. HDL concentration was determined by immunoinhibition and LDL was calculated using the Friedewald formula [21]. All reagents employed in the biochemical tests were obtained from Randox Laboratories (Randox, Antrim, UK).

### Statistical analysis

All values were expressed as mean ± standard deviation (mean ± SD). Statistical tests were processed using STATISTICA 10 software (Stat Soft, Maisons-Alfort, France). The Shapiro-Wilk test revealed that the data were normally distributed. Anthropometric and physical characteristics were compared using Student’s t-test for independent samples. Biochemical, metabolic and physical performance parameters were analyzed using a mixed 2-way ANOVA for independent samples (i.e., MEL and PLA groups) with repeated measures for the TC factor. This factor was “within-subjects” by definition and included two “levels” (Before/After). When appropriate, significant differences among means were tested using Tukey’s honestly significant difference post-hoc test.

Effect sizes were calculated as partial eta-squared (η^2^_p_) for the ANOVA analysis to estimate the meaningfulness of significant findings. η^2^_p_ values of 0.01, 0.06 and 0.13 represent small, moderate, and large effect sizes, respectively [22]. The effect size (d) of changes in each measured variable is defined as the difference between the means divided by the common standard deviation. Threshold values for the interpretation of “d” were 0 to <0.20 (trivial), 0.20 to <0.50 (small), 0.50 to <0.80 (medium) and ≥0.80 (large) [23]. The percentages of changes were calculated according to the following formula: ((V2-V1)/V1) * 100 with V1 being the initial value and V2 being the value after change.

A p-level of 0.05 was selected as the criterion for statistical significance.

## Results

### Biochemical parameters

Statistical analysis showed a significant effect of group, TC and group × TC interaction for AOPP (Table 2). The post hoc test showed a significant increase (~ 26.67%) of AOPP levels after the TC only in the PLA group (p<0.05, d = 2.70). In addition, AOPP levels after the TC in the MEL group became lower (~ 26.67%) compared to the PLA group (p<0.05, d = 2.43).

**Table 2:** Biochemical parameters measured before (BTC) and after (ATC) the training camp (TC) following melatonin (MEL) or placebo (PLA) ingestion.

	PLA		MEL		ANOVA		
	BTC	ATC	BTC	ATC	Group	TC	Interaction
**AOPP (nmol/ mg protein)**	0.30 ± 0.02	0.38 ± 0.04 ^a^	0.32 ± 0.05	0.30 ± 0.03 ^b^	**F**_**(1.19)**_ **= 6.78; p<0.05;** **η**^**2**^_**p**_ **= 0.27**	**F**_**(1,19)**_ **= 8.68; p<0.05; η**^**2**^_**p**_ **= 0.33**	**F**_**(1,19)**_ **= 26.60; p<0.001; η**^**2**^_**p**_ **= 0.60**
**SOD (U/g protein)**	774.82 ± 46.56	744.89 ± 41.49	779.93 ± 55.09	841.42 ± 46.50 ^a^ ^b^	**F**_**(1.19)**_ **= 7.40; p<0.05;** **η**^**2**^_**p**_ **= 0.30**	F_(1.19)_ = 2.36; p>0.05; η^2^_p_ = 0.12	**F**_**(1.19)**_ **= 19.80; p<0.001;** **η**^**2**^_**p**_ **= 0.52**
**CK (IU/l)**	194.30 ± 52.45	273.30 ± 92.25^a^	180.40 ± 59.83	183.67 ± 58.02 ^b^	F_(1.19)_ = 3.60; p>0.05; η^2^_p_ = 0.17	**F**_**(1.19)**_ **= 10.09; p<0.05;** **η**^**2**^_**p**_ **= 0.36**	**F**_**(1.19)**_ **= 8.55; p<0.001;** **η**^**2**^_**p**_ **= 0.32**
**GGT (IU/l)**	11.40 ± 1.17	15.10 ± 1.37 ^a^	12.10 ± 1.45	13.30 ± 2.50	F_(1.19)_ = 0.67; p>0.05; η^2^_p_ = 0.04	**F**_**(1.19)**_ **= 49.45; p<0.001;** **η**^**2**^_**p**_ **= 0.73**	**F**_**(1.19)**_ **= 12.87; p<0.05;** **η**^**2**^_**p**_ **= 0.42**
**AP (IU/l)**	73.90 ± 2.28	80.40 ± 4.12 ^a^	76.60 ± 11.47	82.20 ± 9.67 ^a^	F_(1.19)_ = 0.45; p>0.05; η^2^_p_ = 0.02	**F**_**(1.19)**_ **= 32.07; p<0.001;** **η**^**2**^_**p**_ **= 0.64**	F_(1.19)_ = 0.12; p>0.05; η^2^_p_ = 0.01

AOPP: Advanced oxidation protein product; SOD: Superoxide dismutase; CK: Creatine kinase; GGT: Gamma-glutamyltransferase; AP: Alkaline phosphatase.

^a^ Significant difference in comparison with values measured before the TC.

^b^ Significant difference in comparison with values measured in PLA group.

Moreover, the statistical analysis showed a significant effect of group and group × TC interaction for SOD (Table 2). After the TC, the post hoc test showed a significant increase (~ 7.88%) in SOD activity in the MEL group (p<0.001, d = 1.21). In addition, SOD activity became higher (~ 12.96%) compared to the PLA group (p<0.001, d = 2.19).

Regarding CK and GGT, statistical analysis showed a significant effect of TC and a group × TC interaction (Table 2). The post hoc test showed a significant increase in CK (~ 40.66%) and GGT (~ 32.46%) activities only in the PLA group (p<0.05, d = 1.09 for CK and p<0.001, d = 2.91 for GGT) after the TC. In addition, CK activity after the TC in the MEL group became lower (~ 32.80%) compared to the PLA group (p<0.05, d = 1.19). Moreover, a significant TC effect was revealed for AP (Table 2) and the post hoc test showed a significant increase in AP activity after the TC in both groups (p<0.05, d = 2.03, rate of increase ~ 8.80% and p<0.05, d = 0.53, rate of increase ~ 7.31% for PLA and MEL group respectively).

### Metabolic parameters

Statistical analysis showed a significant group × TC interaction for Cre, a significant TC effect for Cre, Ur, HDL and Gl and a significant group effect for Ur, TP, HDL and Tg (Table 3). The post hoc analysis demonstrated a significant increase (~ 14.14%) in Cre level following the TC only in the PLA group (p<0.001, d = 1.33) and a significant increase in Ur levels in MEL (p<0.05, d = 1.40, rate of increase ~ 41%) and PLA (p<0.001, d = 1.97, rate of increase ~ 43.22%) groups. However, no significant effect was revealed for TP, TCh, LDL, Tg and Gl levels.

**Table 3:** Metabolic parameters measured before (BTC) and after (ATC) the training camp (TC) following melatonin (MEL) or placebo (PLA) ingestion.

	PLA		MEL		ANOVA		
	BTC	ATC	BTC	ATC	Group	TC	Interaction
**Cre (μmol/l)**	68.87 ± 7.91	78.61 ± 6.77 ^a^	72.77 ± 5.50	73.20 ± 4.98	F_(1.19)_ = 0.09; p>0.05; η^2^_p_ = 0.01	**F**_**(1.19)**_ **= 13.80; p<0.05;** **η**^**2**^_**p**_ **= 0.43**	**F**_**(1.19)**_ **= 11.54; p<0.05;** **η**^**2**^_**p**_ **= 0.39**
**Ur (mmol/l)**	3.54 ± 0.79	5.07 ± 0.77 ^a^	3.00 ± 0.88	4.23 ± 0.88 ^a^	**F**_**(1.19)**_ **= 4.76; p<0.05;** **η**^**2**^_**p**_ **= 0.21**	**F**_**(1,19)**_ **= 51.31; p<0.001; η**^**2**^_**p**_ **= 0.74**	F_(1,19)_ = 0.61; p>0.05; η^2^_p_ = 0.03
**TP (g/l)**	70.94 ± 1.97	70.64 ± 3.83	74.40 ± 4.88	74.23 ± 5.08	**F**_**(1.19)**_ **= 6.22; p<0.05;** **η**^**2**^_**p**_ **= 0.26**	F_(1.19)_ = 0.04; p>0.05; η^2^_p_ = 0.00	F_(1.19)_ = 0.00; p>0.05; η^2^_p_ = 0.00
**TCh (mmol/l)**	3.37 ± 0.28	3.26 ± 0.45	3.56 ± 0.51	3.53 ± 0.77	F_(1.19)_ = 1.11; p>0.05; η^2^_p_ = 0.06	F_(1.19)_ = 0.47; p>0.05; η^2^_p_ = 0.03	F_(1.19)_ = 0.16; p>0.05; η^2^_p_ = 0.01
**HDL (mmol/l)**	1.02 ± 0.18	1.12 ± 0.24	1.29 ± 0.23	1.39 ± 0.30	**F**_**(1.19)**_ **= 6.86; p<0.05;** **η**^**2**^_**p**_ **= 0.28**	**F**_**(1.19)**_ **= 8.25; p<0.05;** **η**^**2**^_**p**_ **= 0.31**	F_(1.19)_ = 0.02; p>0.05; η^2^_p_ = 0.00
**LDL (mmol/l)**	1.91 ± 0.26	1.77 ± 0.33	1.73 ± 0.44	1.65 ± 0.54	F_(1.19)_ = 0.79; p>0.05; η^2^_p_ = 0.04	F_(1.19)_ = 2.80; p>0.05; η^2^_p_ = 0.13	F_(1.19)_ = 0.16; p>0.05; η^2^_p_ = 0.01
**Tg (mmol/l)**	0.97 ± 0.30	0.87 ± 0.17	1.20 ± 0.33	1.08 ± 0.14	**F**_**(1.19)**_ **= 6.89; p<0.05;** **η**^**2**^_**p**_ **= 0.28**	F_(1.19)_ = 2.52; p>0.05; η^2^_p_ = 0.12	F_(1.19)_ = 0.01; p>0.05; η^2^_p_ = 0.00
**Gl (mmol/l)**	4.30 ± 0.55	4.06 ± 0.52	4.29 ± 0.53	4.09 ± 0.48	F_(1.19)_ = 0.00; p>0.05; η^2^_p_ = 0.00	**F**_**(1.19)**_ **= 5.03; p<0.05;** **η**^**2**^_**p**_ **= 0.22**	F_(1.19)_ = 0.04; p>0.05; η^2^_p_ = 0.00

Cre: Creatinine; Ur: Urea; TP: Total protein; TCh: Total cholesterol; HDL: High-density lipoprotein; LDL; low-density lipoprotein; Tg: Triglyceride; Gl: Glucose.

^a^ Significant difference in comparison with values measured before the TC.

### Physical performance

Statistical analysis showed a significant effect of TC and group × TC interactions for SJ, CMJ, 5-JT, MAT and 20m-Sp. In addition, a significant group effect was revealed for 20m-Sp (Table 4). The post hoc test showed, in both groups, a significant decrease in SJ (p<0.001, d = 0.53, rate of decrease ~ -7.22% and p<0.001, d = 1.82, rate of decrease ~ -14.84% respectively for MEL and PLA groups), MAT (p<0.001, d = 4.71, rate of decrease ~ -13.49% and p<0.001, d = 3, rate of decrease ~ -19.16% respectively for MEL and PLA groups) and 20m-Sp performances (p<0.001, d = 4.65, rate of decrease ~ -15.63% and p<0.001, d = 11.35, rate of decrease ~ -25.87% respectively for MEL and PLA groups). In addition, after the TC, 20m-Sp performance in the PLA group became lower (~ 7.84%) compared to the MEL group (p<0.001, d = 2.93). Moreover, CMJ and 5-JT performances decreased after the TC only in the PLA group (p<0.001, d = 0.87, rate of decrease ~ -7.68% and p<0.001, d = 1, rate of decrease ~ -4.26% respectively).

**Table 4:** Physical performance measured before (BTC) and after (ATC) the training camp (TC) following melatonin (MEL) or placebo (PLA) ingestion.

	PLA		MEL		ANOVA		
	BTC	ATC	BTC	ATC	Group	TC	Interaction
**SJ (cm)**	34.63 ± 2.79	29.49 ± 2.86 ^a^	34.91 ± 4.81	32.39 ± 4.64 ^a^	F_(1.19)_ = 0.87; p>0.05; η^2^_p_ = 0.05	**F**_**(1.19)**_ **= 103.26; p<0.05;** **η**^**2**^_**p**_ **= 0.85**	**F**_**(1.19)**_ **= 12.08; p<0.05;** **η**^**2**^_**p**_ **= 0.40**
**CMJ (cm)**	35.40 ± 3.53	32.68 ± 2.69 ^a^	36.02 ± 2.61	34.97 ± 2.96	F_(1.19)_ = 1.16; p>0.05; η^2^_p_ = 0.06	**F**_**(1,19)**_ **= 24.18; p<0.001; η**^**2**^_**p**_ **= 0.59**	**F**_**(1,19)**_ **= 4.75; p<0.05;** **η**^**2**^_**p**_ **= 0.22**
**5-JT (m)**	12.21 ± 0.47	11.69 ± 0.56 ^a^	12.46 ± 0.70	12.32 ± 0.64	F_(1.19)_ = 2.78; p>0.05; η^2^_p_ = 0.13	**F**_**(1.19)**_ **= 40.83; p<0.001;** **η**^**2**^_**p**_ **= 0.69**	**F**_**(1.19)**_ **= 13.53; p<0.05;** **η**^**2**^_**p**_ **= 0.43**
**MAT (s)**	5.74 ± 0.30	6.84 ± 0.43 ^a^	5.78 ± 0.19	6.56 ± 0.14 ^a^	F_(1.19)_ = 0.89; p>0.05; η^2^_p_ = 0.05	**F**_**(1.19)**_ **= 827.21; p<0.001;** **η**^**2**^_**p**_ **= 0.98**	**F**_**(1.19)**_ **= 24.24; p<0.001;** **η**^**2**^_**p**_ **= 0.57**
**20m-Sp (s)**	3.17 ± 0.04	3.99 ± 0.10 ^a^	3.20 ± 0.12	3.70 ± 0.09 ^a^ ^b^	**F**_**(1.19**_ **= 12.41; p<0.05;** **η**^**2**^_**p**_ **= 0.41**	**F**_**(1.19)**_ **= 913.48; p<0.001;** **η**^**2**^_**p**_ **= 0.98**	**F**_**(1.19)**_ **= 54.43; p<0.001;** **η**^**2**^_**p**_ **= 0.75**

SJ: Squat jump; CMJ: Countermovement jump; 5-JT: Five-jump test; MAT: Modified agility T-test; 20m-Sp: 20-m sprint.

^a^ Significant difference in comparison with values measured before the TC.

^b^ Significant difference in comparison with values measured in PLA group.

## Discussion

This research aimed to examine the effect of MEL ingestion on physical performance decline and biochemical and metabolic responses during an intensive TC for soccer players. This study showed that the training period induced an increased oxidative stress state. AOPP, an important marker of proteins oxidation [18], were significantly higher in the blood of PLA group players. This raised oxidative stress state was also observed in response to football training [10]. The increase in oxidative stress level in response to exhaustive training may decrease muscle force generation [24]. Therefore, an effective and balanced antioxidant defense system is crucial for the prevention of ROS damage. Appositely, we observed that MEL ingestion prevented an upsurge of AOPP level. Likewise, previous investigations recommended MEL to counteract exercise-induced oxidative stress [1,8,10,25]. This may be explained by the extremely important antioxidant action of MEL and its metabolites produced as a result of the neutralization of ROS [9]. The mechanism by which MEL and its metabolites counteract ROS is referred to the free radical scavenging cascade and differentiates MEL from other antioxidants [9,26]. In addition, the amphiphilic nature of MEL allows it to cross all intercellular barriers and exert its antioxidant effect in all milieus [25,26]. This greatly increases the effectiveness of MEL as a defender against ROS.

After the TC, SOD activity increased in the MEL group. Accordingly, previous studies showed that MEL ingestion amplified the activity of antioxidant enzymes [1,10]. MEL is an endogenous antioxidant but also acts as an indirect free radical scavenger by activating the main antioxidant enzymes [25,27]. The indirect antioxidant activity of MEL is associated with stimulation of the major antioxidant enzymes via the regulation of enzyme activity or gene expression [10,27,28]. MEL reduces the half-life of mRNAs coding for SOD, and in this case induces more available levels of mRNAs with shorter half-lives [28]. SOD serves as a frontline fighter against ROS oxidative assault, and activity of this enzyme has an essential protective role in reducing molecular damage [28]. In addition, MEL is involved in maintaining elevated intracellular concentrations of reduced GSH, as this indoleamine induces activity of the rate-limiting enzyme of GSH synthesis [27].

The results of the present study showed that the CK activity increased in the PLA group after the TC, confirming an increase in muscle damage. Likewise, previous investigations revealed an important alteration in muscle damage biomarkers in response to soccer matches and training [8,29]. However, CK activity did not rise in the MEL group. These results revealed less muscle damage and may indicate a protective effect of MEL on skeletal muscles during an intensive training period. This may help athletes to train better, since it has been shown that an elevated activity of these enzymes was correlated with muscle cramps [14]. Accordingly, previous studies have found an important decrease in muscle injury markers after MEL ingestion [1,8,25]. It also enhanced muscle healing and regeneration in rats suffering from muscle injury [11]. MEL may be considered as a promising therapy for trauma/sports related muscle injury [30]. It inhibits nuclear factor kappa B (NFκB), reduces cytokine expression, and increases protein kinase B (PKB), which downregulates the ratio of muscle-specific RING finger protein 1 (MURF-1) and muscle atrophy F-box (MAF_BX_) in order to reduce the extent of muscle injury and expedite post-injury muscle recovery [30].

GGT is a marker of general liver health in medicine. It serves as a common index for the detection of liver injury [31]. In the present study, the results showed that GGT activity increased after the TC in the PLA group, indicating an increase in liver damage and reflecting the great challenge caused by the training. Similarly, prior research has shown that GGT activity increased after strenuous exercise [14]. The rise in this enzyme is linked to exercise intensity and duration [14]. The alteration in hepatic function caused by exercise is related to changes in liver cell membrane owing to lipid peroxidation induced by impaired blood flow and the production of free radicals [32].

However, MEL ingestion in this study reduced the GGT activity after the TC, indicating less liver damage. This is the first study showing an effect of MEL ingestion on GGT activity in response to physical exercise. The role of GGT in exercise is counteracting ROS by breaking down extracellular glutathione and making its component amino acids available to cells for repair [30,32,33]. Therefore, after MEL ingestion, there would be a lower need of GGT given the radical scavenging and antioxidant effects of MEL. Clinical studies showed that MEL displayed protective effects in liver injuries and diseases by inhibiting liver neutrophil infiltration, necrosis and apoptosis, improving mitochondrial physiology, preventing oxidative damage, reducing the severity of morphological alterations and suppressing liver fibrosis [34].

Moreover, the results of the present study showed that AP activity increased after the TC in MEL and PLA groups. Accordingly, prior research has shown that AP activity is related to bone activity [35] and increased after high-intensity, low-impact exercise [36]. AP is an enzyme found mainly in bone and liver, which is involved in both removal of mineral phosphate from molecules and inflammatory conditions [35].

All the above biochemical parameters are known as markers of muscle fatigue and injuries due to exercise [14]. Therefore, the reducing effect of MEL on these biomarkers may help athletes during congested schedules to recover faster and to reduce the risk of injuries.

Cre and Ur serum concentrations are the most widely used and commonly accepted measures for renal function in clinical medicine. Cre is used in sports medicine to assess the athlete’s general health status, particularly in events where hydro-electrolytic balance is important [14]. Aerobic or strength exercise was associated with high levels of these biomarkers [37]. Accordingly, in the present study, the TC resulted in greater levels of Cre and Ur. However, MEL ingestion showed a protective effect on renal function and decreased Cre level after the TC. Similarly, Leonardo-Mendonça et al. [38] reported a reduction in Cre level after MEL ingestion during an intensive training period.

Otherwise, the main results of the present study show that the decline in physical performance (i.e., CJ, 5-JT, 20m-Sp) in soccer players after attending an intensive TC is attenuated by MEL. This finding may be explained by the decrease of cellular damage after several days of MEL supplementation, since it has been shown that high levels of ROS promote contractile dysfunction, resulting in muscle weakness and fatigue [24]. Indeed, several studies have reported that antioxidant supplementation reduces muscle damage and oxidative stress [39], which can reduce fatigue [40] and expedite recovery of muscle function [39].

After the training period, MEL group athletes have better physical performance (i.e., 20m-Sp) than PLA group athletes. This is due to greater deterioration of the performance of PLA group rather than improvement of the performance of the MEL group. In line with this, a previous study [13] showed improved physical performance and decreased perception of fatigue in response to acute administration of MEL after strenuous late evening exercise. This was explained by MEL-induced improvement in sleep quality and quantity. The regulation of circadian rhythms via MEL is crucial for the optimal generalized physiology of organisms and for the microphysiology of molecular functions [7]. The improvement of the restoration process during sleep after MEL ingestion may modulate oxidative and immunology processes [7,10]. So, in the present study, a possible positive effect of the nocturnal MEL ingestion on sleep may also explain the amelioration of physical performance decline.

However, previous investigations did not show any impact of diurnal MEL ingestion on short-term physical performance after an exhaustive training session [16,41]. In these studies, a single dose of 6 mg of MEL was provided before the training session, whereas in the present study, a daily dose of 5 mg of MEL was applied throughout the training period.

There are some limitations of this study. We did not assess the possible effect of MEL on sleep quality and quantity during the TC. In addition, we overlooked the assessment of delayed biochemical responses. Previous investigations have found that markers of muscle and liver damage remain elevated for many days after exhaustive exercise [42]. Furthermore, the small size of the sample limits the generalization of the present results. Further investigations are required to generalize the findings of this study to other populations with different sexes, from different age categories and practicing other sports.

## Conclusion

The present study showed that MEL intake during an intensive training period exerted beneficial effects on oxidative stress, antioxidant status and muscle damage. In addition, it prevented the increase of biomarkers of renal function and liver damage. Furthermore, it attenuated the decline of short term physical performance. Therefore, MEL is a suitable supplement, which can help soccer players to improve recovery because of its well-known antioxidant and protective effects.

## Supporting information

S1 File (XLSX)
